# Fluidic Logic Used in a Systems Approach to Enable Integrated Single-Cell Functional Analysis

**DOI:** 10.3389/fbioe.2016.00070

**Published:** 2016-09-21

**Authors:** Naveen Ramalingam, Brian Fowler, Lukasz Szpankowski, Anne A. Leyrat, Kyle Hukari, Myo Thu Maung, Wiganda Yorza, Michael Norris, Chris Cesar, Joe Shuga, Michael L. Gonzales, Chad D. Sanada, Xiaohui Wang, Rudy Yeung, Win Hwang, Justin Axsom, Naga Sai Gopi Krishna Devaraju, Ninez Delos Angeles, Cassandra Greene, Ming-Fang Zhou, Eng-Seng Ong, Chang-Chee Poh, Marcos Lam, Henry Choi, Zaw Htoo, Leo Lee, Chee-Sing Chin, Zhong-Wei Shen, Chong T. Lu, Ilona Holcomb, Aik Ooi, Craig Stolarczyk, Tony Shuga, Kenneth J. Livak, Cate Larsen, Marc Unger, Jay A. A. West

**Affiliations:** ^1^New Technologies Research Department, Fluidigm Corporation, South San Francisco, CA, USA

**Keywords:** single-cell, mRNA-seq, functional studies, Fluidigm, Polaris

## Abstract

The study of single cells has evolved over the past several years to include expression and genomic analysis of an increasing number of single cells. Several studies have demonstrated wide spread variation and heterogeneity within cell populations of similar phenotype. While the characterization of these populations will likely set the foundation for our understanding of genomic- and expression-based diversity, it will not be able to link the functional differences of a single cell to its underlying genomic structure and activity. Currently, it is difficult to perturb single cells in a controlled environment, monitor and measure the response due to perturbation, and link these response measurements to downstream genomic and transcriptomic analysis. In order to address this challenge, we developed a platform to integrate and miniaturize many of the experimental steps required to study single-cell function. The heart of this platform is an elastomer-based integrated fluidic circuit that uses fluidic logic to select and sequester specific single cells based on a phenotypic trait for downstream experimentation. Experiments with sequestered cells that have been performed include on-chip culture, exposure to various stimulants, and post-exposure image-based response analysis, followed by preparation of the mRNA transcriptome for massively parallel sequencing analysis. The flexible system embodies experimental design and execution that enable routine functional studies of single cells.

## Introduction

Recent single-cell transcriptomic analyses have documented the importance of cellular heterogeneity in studying cancer (Ennen et al., 2014; Saadatpour et al., 2014; Kim et al., 2015), immunology (Shalek et al., 2014), developmental biology (Briggs et al., 2015), stem cell research (Wilson et al., 2015), and neurobiology (Pollen et al., 2015). It has been estimated that the human body contains 37.2 trillion cells (Bianconi et al., 2013), excluding the complex microbiome that lives in the human body. High-throughput single-cell mRNA sequencing provides an unbiased path to classifying this vast number of cells into cell types. This endeavor has stimulated the development of methods to increase throughput (Fan et al., 2015; Klein et al., 2015; Macosko et al., 2015). The classification of cell types can be thought of as a high-resolution anatomy. At the single-cell level, moving from anatomy to physiology or from description to mechanism means moving from cell type to cell function. This will require integrating transcriptional data with other cellular measurements. In this regard, progress has been made in obtaining transcriptomic and genomic information (Dey et al., 2015; Macaulay, 2015), transcriptomic and epigenomic information (Angermueller et al., 2016), or transcriptomic and proteomic information (Darmanis et al., 2016; Frei et al., 2016) from the same single cell.

Moving from cell type to cell function will also require understanding how single-cell profiles change in response to perturbations. It is important to examine these effects at the single-cell level because cell-to-cell heterogeneity has been observed in a diverse set of circumstances, such as the response of macrophages to bacterial invasion (Avraham et al., 2015), the response of hematopoietic cells to various drugs (Bendall et al., 2011), and drug resistance in adenocarcinoma cells (Kim et al., 2015). Progress in the long-term culture of circulating tumor cells (Gao et al., 2014; Yu et al., 2014; Cayrefourcq et al., 2015; Alix-Panabières et al., 2016) enables single-cell functional studies on this important class of cells, which should lead to improved cancer diagnosis and therapy. Performing perturbation experiments on single cells requires care in maintaining the appropriate microenvironment. Examining the effects of serum on mouse embryonic stem cells (ESCs), researchers (Guo et al., 2016) concluded that “a large proportion of intracellular network variability is due to the extracellular culture environment.” Microfluidic-based approaches are attractive for the precise control of the microenvironment because they enable structures at a size appropriate for single cells. Microfluidic systems for high-throughput preparation of sequencing libraries, though, have cell lysis as the initial step and thus are not suitable to maintain single cells for experimentation. What is required is a system specifically designed to capture, maintain, perturb, and observe single cells and then prepare these cells for high-dimensional analysis.

**Figure 1:**
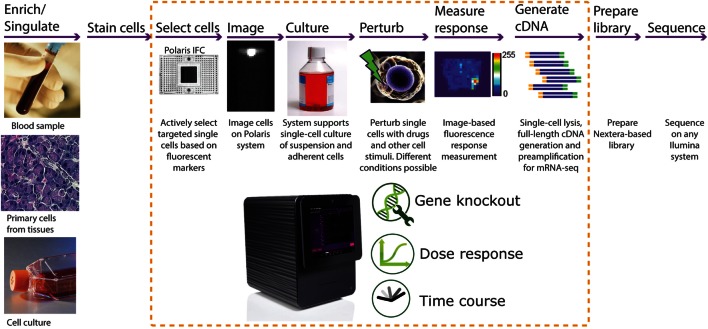
**Typical workflow for single-cell functional studies**. The input single-cell suspension can be obtained from blood, primary cells, or cell culture. In the case of rare cells or a subset of cell population, there is an option to enrich them prior to use with Polaris using either fluorescence-assisted cell sorting (FACS) or other methods. Subsequently, the cells are labeled with a universal fluorescent marker for tracking the cells on the Fluidigm^®^ Polaris system. Most of the single-cell functional study steps are automated on the Polaris system. The Polaris system generates preamplified full-length cDNA, which can be further processed for library preparation and massive parallel sequencing for mRNA sequencing.

In this paper, we report development of an integrated fluidic circuit (IFC) that uses fluidic logic to actively select and sequester desired single cells based on particular biological markers of interest. This Polaris™ IFC can sequester up to 48 single cells. If required, the cells can be cultured in appropriate medium in order to control and manipulate the microenvironment around the sequestered cells. For adherent cells, appropriate extracellular matrix (ECM) can be coated inside the culture chambers. The single cells can be perturbed with a drug or other stimuli (i.e., mRNA, cytokines, bacteria, or viruses), with the response to perturbation monitored and measured by fluorescence imaging. Subsequently, the single cells are processed for cell lysis, reverse transcription (RT), and full-length transcriptome amplification using template-switching chemistry. Following harvest from the IFC, sequencing libraries are generated using a modified Nextera^®^ protocol and sequenced on any Illumina^®^ platform (Figure 1).

## Materials and Methods

### Design and Fabrication of Logic-Based Integrated Fluidic Circuit

**Figure 2:**
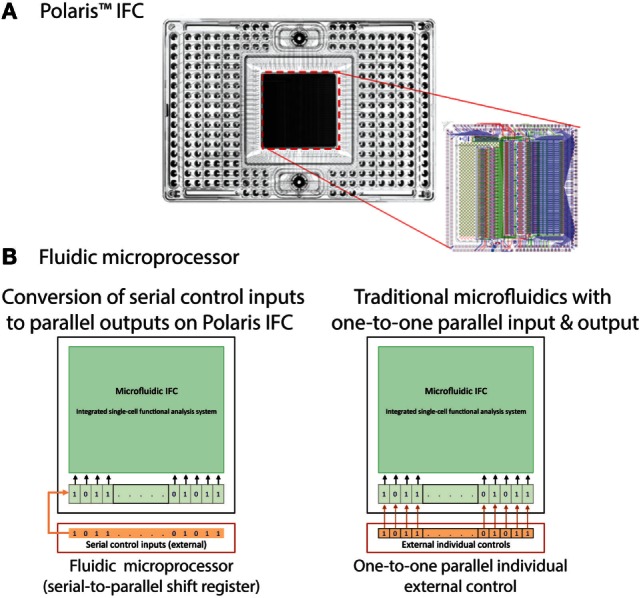
**(A)** Polaris mRNA-seq dosing integrated fluidic circuit (IFC). The polydimethylsiloxane (PDMS) elastomer is attached to a plastic carrier. In order to image the cells with low-fluorescence background on the Polaris system, the PDMS is backed with a black material. This black backing is removed after cell perturbation and fluorescence response measurement. Removal of black backing enables high-resolution imaging of cells on a fluorescent microscope. The CAD drawing of the microfluidic components is shown on the right. The green channels are the control lines. **(B)** Polaris IFC uses fluidic processor to receive serial control inputs and converts them to parallel shift register elements. Traditional microfluidic control elements use one external control for every internal control.

The nanoscale IFC consists of a plastic carrier and a polydimethylsiloxane (PDMS) core (Figure 2A) or fluidic circuit. The carrier contains reservoir wells for input and output of reagents and circuit control. It provides a platform to facilitate interfacing with the fluidic circuit. The fluidic circuit with the desired microfluidic control components was fabricated using multilayer soft lithography (MSL^®^) process (Unger et al., 2000). Fluidic circuit components include flow and control channels, valves, multiplexors, and logic devices [such as serial-to-parallel shift register (SR)]. Fabrication and operational details of the fluidic logic circuits and devices were reported earlier (Devaraju and Unger, 2012). The IFC is designed to have the capability to actively select single cells based on fluorescent markers, isolate them to a desired holding location (cell capture site), apply individual conditions (feed medium and dose reagents to cells), and finally study the functional response. Execution of all these complex functions in a routine fashion requires flexible, programmable operational control, which in turn requires many controls in a parallel manner. Traditionally, in microfluidics, a dedicated external control line is required to independently control a set of valves. This imposes a limitation on the number of practical on-chip control operations and poses a challenge for scalability by requiring more external hardware. On-chip control architecture capable of receiving and processing data by elementary computation and decision-making can integrate programmability of controls on-chip and allows an increase in the number of on-chip control lines for the same number of external chip connections (Figure 2B). We developed such a microfluidic fluidic logic and implemented it on our Polaris IFC.

The state-based microfluidic fluidic logic devices and circuits utilize static gain and normally closed valves (NCVs). NCVs are fabricated by filling specialized control channels with a flash curable prepolymer and curing while the valve is closed. The resulting closed valve exerts certain force against fluidic pressure to keep the valve closed. The valves are characterized by breakthrough pressure: the threshold pressure in the flow channel required to push open the valve and restore the continuity of the flow. Breakthrough pressure for an NCV can be tailored by controlling the pressure at which they are cured. Using these NCVs, we have developed static gain valves (SGV) that have the ability to control higher (or equal) fluidic pressure using a lower pressure. This type of valve is essential to create any logic/feedback structures (to account for signal strength losses), which can receive the output of the previous element/gate and use it as an input for decision making.

**Figure 3:**
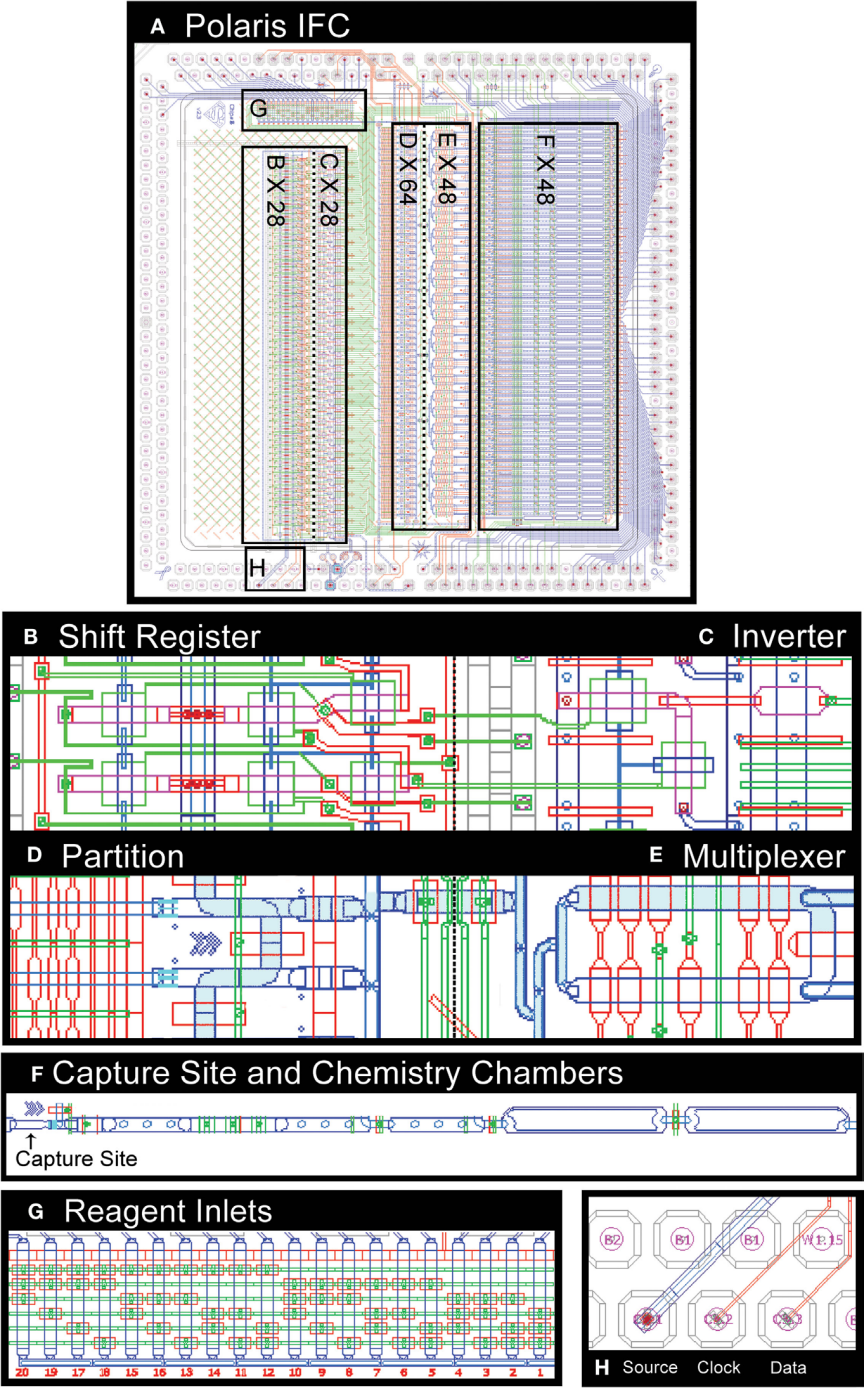
**CAD drawing of microfluidic control components on Polaris mRNA-seq dosing IFC (A)**. The shift register **(B)** enables active selection of single cells. The dilute single-cell suspension is loaded into a serpentine partition channel **(D)**. The cell suspension liquid flow is stopped, and the partition channel is imaged to identify single cells based on a particular set of fluorescence markers. The selected cells are then microfluidically moved downstream to a cell capture site **(F)** through a multiplexer **(E)**. The IFC is capable of accepting 20 reagents **(G)** as input. The shift register uses inverter **(C)** and a set of source, clock, and data **(H)**.

Utilizing the SGV, we next built an inverter (NOT gate), which was further used to build more complex circuits including bi-stable flip flops, clocked flip flops (latches), delay flip flops (D-FF, one bit of the SR), and complex microprocessors (SR). A SR that is capable of processing *n* + 1 bits of data is formed by combining *n* D flip flops (bits of SR). The SR presented here uses air as the medium and receives three active high-pressure inputs: source, clock, and data (Figure 3). The pneumatic output of the SR cannot be used to control the flow of liquids in microchannels directly, due to risk of introducing bubbles. In order to address this issue, the signal medium is converted from air to liquid using an inverter.

The Polaris IFC microprocessor receives 28 external signals serially and processes them into 28 parallel independent controls capable of controlling individual valves or a set of valves. Five dedicated high-pressure external active signals are required for a SR. The CAD drawing of the various microfluidic components on a Polaris IFC is shown in Figure 3. The IFC can accept up to 20 independent reagents. The fluorescently labeled cells are loaded in a serpentine partition channel. Based on a desired combination of up to three fluorescent markers (refer to Section “Polaris Instrument Design” for excitation and emission details), single cells are selected and sequentially isolated to the cell capture sites through a multiplexer. Up to 48 single cells can be sequestered on a single Polaris IFC. Subsequently, these 48 cells are processed through template-switching chemistry for full-length cDNA generation for mRNA-seq. In brief, the cells are lysed and reverse-transcribed, and full-length cDNA is preamplified by long and accurate PCR.

### Polaris Instrument Design

**Figure 4:**
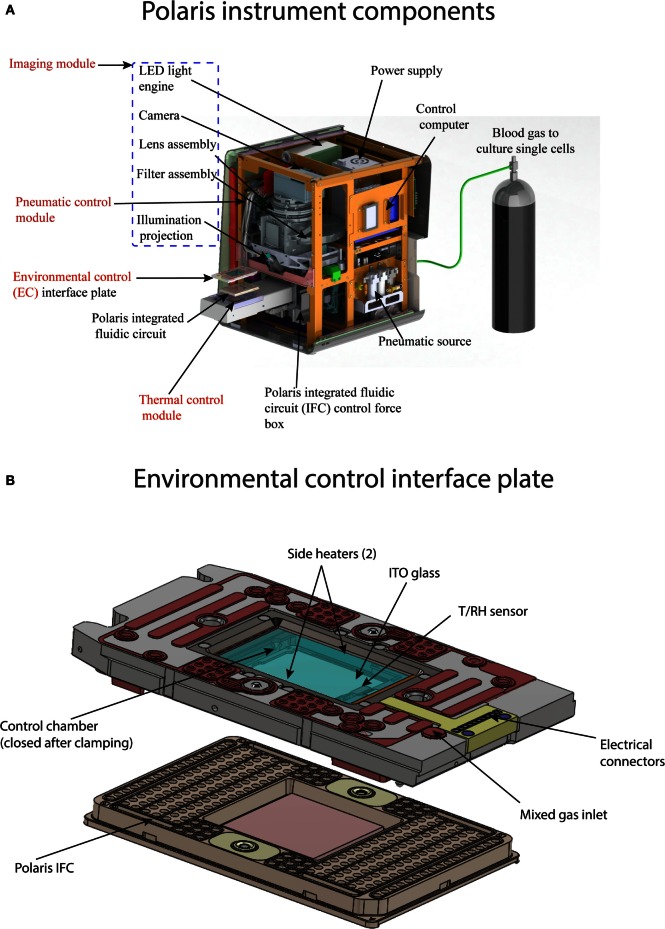
**(A)** Components of Polaris instrument. The instrument consist of four major modules: (1) thermal module, enables preparative chemistry on sequestered single cells; (2) imaging module, consists of LED excitation and emission collection by a camera; (3) pneumatic module, controls the movement of reagents inside microfluidic channel by application of positive pressure on the IFC carrier; and (4) environmental control module, maintains the temperature, humidity, and blood gas flow rate for single-cell culture on-IFC. **(B)** Components of environmental control interface plate. The top of the interface plate contains glass coated with indium tin oxide. Internal heaters are used to maintain the temperature of the closed chamber between the interface plate and Polaris IFC. The temperature and relative humidity inside the closed chamber (after clamping with Polaris IFC) are measured by T/RH sensor. Blood or premixed gas required for cell culture is pumped through mixed gas inlet port on the interface plate. Polaris IFC is shown for reference.

The Fluidigm Polaris system (Figure 4A) consists of four major modules: (1) thermal control module; (2) imaging module; (3) pneumatic control module; and (4) environmental control (EC) module. The thermal module consists of a Peltier-based thermoelectric couple (TEC) device for heating/cooling. The TEC module can provide temperature in the range of 4–99°C. Vacuum grooves on the thermal module are designed to enable tight contact with the glass-based integrated heat spreader (IHS) on the Polaris IFC. This ensures thermal uniformity across the fluidic circuit. The imaging module contains a five-color LED light engine for excitation (Ex wavelengths: 438, 475, 530, 575, and 632 nm). The light source from the engine is collected and projected onto the fluidic circuit using fiber optics. The emitted signal from the fluidic circuit passes through an emission filter (five Em wavelengths: 488, 525, 570, 630, and 700 nm) and is collected by CCD camera with 6-μm pixel resolution through a custom-designed collimator lens.

The pneumatic control module generates and stores air with volume up to 1 L. The system can achieve a maximum pressure of 100 psi. The pneumatic controller generates a vacuum on the thermal chuck, clamps the Polaris IFC against the EC interface plate (IP) to enable a closed environment around the IFC, and loads reagents from the inlets on the IFC carrier to the microchannels and reagent chambers of the fluidic circuit. The system contains segregated zones to regulate four different pressures simultaneously. The EC module provides an environment suitable for cell culture using user-desired gas composition. Environmental parameters such as temperature, relative humidity (RH), and mix gas flow rate across the fluidic circuit are monitored and controlled. The gas inside the closed chamber is heated by two heater coils. The gas inlet on the EC IP is used to regulate the flow of gas across the fluidic circuit. The EC IP (Figure 4B) contains an indium tin oxide (ITO) coated glass on the top to maintain thermal control in the EC while allowing imaging through the EC IP. During cell culture operation, the ITO glass is heated to prevent condensation. During cell culture, the environment around the fluidic circuit is maintained by blood gas (5% CO_2_, 5% oxygen, and 90% nitrogen) or premixed gas of choice (for example, 5% CO_2_, 20% oxygen, and 75% nitrogen). Before on-IFC cell culture, a rectangular sponge saturated with water is installed inside the closed chamber to provide the desired humidity through heating. The EC IP is equipped with two sensors (T/RH) to measure and maintain temperature at 37°C and RH at 90%.

### K562 Cell Culture and CD59 Staining

K562 cells (ATCC^®^ CCL-243) are cultured in T25 flasks in a volume ranging from 10 to 15 mL in an incubator (37°C, 5% CO_2_). The culture medium contains IMDM + GlutaMAX™-I + 25 mM HEPES + 3.024 g/L sodium bicarbonate (Gibco, 31980-030) and is supplemented with 10% FBS. The cells were fed every 2–3 days by dilution to 200,000 cells/mL. The K562 cells were stained with CellTracker™ Orange (CTO) CMRA Dye (Thermo Fisher Scientific, C34551) as universal marker and Alexa Fluor^®^ 647 conjugated CD59 antibody. The recommended dyes and corresponding excitation and emission filters on the Polaris system are shown in Table 1. Immediately before use, the cell staining solution was prepared by adding 0.6 μL of 1 mM CTO to 2 mL of HBSS without calcium or magnesium (−/−) at a final concentration of 0.3 μM. The cell staining solution was protected from light until use within 30 min. A total of ~1.5 × 10^6^ cells was aliquoted in a 15 mL non-pyrogenic conical tube. The cell suspension was centrifuged at 300 × *g* for 3 min. Following this, the medium was aspirated without disturbing the pellet, and 2 mL of cell staining solution was added to the pellet and gently suspended by pipetting up and down three times. The cells were then incubated in the dark at 37°C for 20 min with occasional inverting and flicking. Following this, the cells were washed by adding 12 mL of HBSS to the cells in the 2 mL of staining buffer and then centrifuged at 300 × *g* for 5 min. Supernatant was aspirated and discarded without disturbing the pellet. The pellet was then resuspended in 200 μL of HBSS. The CTO-stained K562 cells were split into two tubes of 100 μL each. One tube was used as negative surface-stained cell population, and the other tube was processed further to stain CD59 epitope. In order to stain the surface CD59 epitope, 10 μL of CD59 biotinylated antibody (BD Biosciences, 555762, 100 tests, 2.0 mL) was added to 100 μL of CTO-stained cells. For negative surface-stained cell control, 10 μL of HBSS was added. Both the tubes were incubated at room temperature for 20 min with occasional inverting and flicking. Subsequently, 13 mL of HBSS was added to each tube and centrifuged at 300 × *g* for 5 min. The pellet was resuspended in 100 μL of HBSS. To this, 0.5 μL of Streptavidin Alexa Fluor^®^ 647 (Thermo Fisher Scientific, S32357, 2 mg/mL stock) was added to positive-stain tube with CD59 biotinylated antibody in 100-μL cell suspension. This solution was mixed gently by pipetting up and down five times. Following this, the stain solution was incubated at room temperature for 15 min with occasional flicking. Again, 13 mL of HBSS was added to each tube, mixed by gently pipetting up and down, and centrifuged at 300 × *g* for 5 min. The supernatant was removed, and the pellet was resuspended in ~100–150 μL culture medium with FBS, but without phenol red, to prevent high background fluorescence during cell selection on the Polaris system. The resuspension volume of culture medium accounts for cell losses during the staining procedure and was chosen to yield a cell concentration greater than the target concentration of 550 cells/μL. Typically, 10 μL of cell mix is loaded into a C-Chip™ Disposable Hemocytometer (INCYTO, DHC-N01) and imaged on the Polaris system to estimate the staining intensity and purity. In order to achieve optimal buoyancy, cells in the range of 333–550 cells/μL are mixed with suspension reagent (Fluidigm, 101-0434). Typically, the ratio of cells to cell suspension reagent is 3:2. However, this ratio might need optimization depending on the cell type.

**Table 1:** **Recommended stains and corresponding excitation and emission filter on Polaris system**.

Channel name	Excitation^a^	Emission	Recommended stains
FAM™	475/40	525/25	Alexa Fluor 488 (selection marker)
VIC^®^	530/20	570/30	CellTracker Orange CMRA Dye (universal marker)
Cy5^®^	632/28	700/30	Alexa Fluor 647 (selection marker)

*^a^Excitation values are center wavelength/band pass (≥90%)*.

### IFC Operation

**Figure 5:**
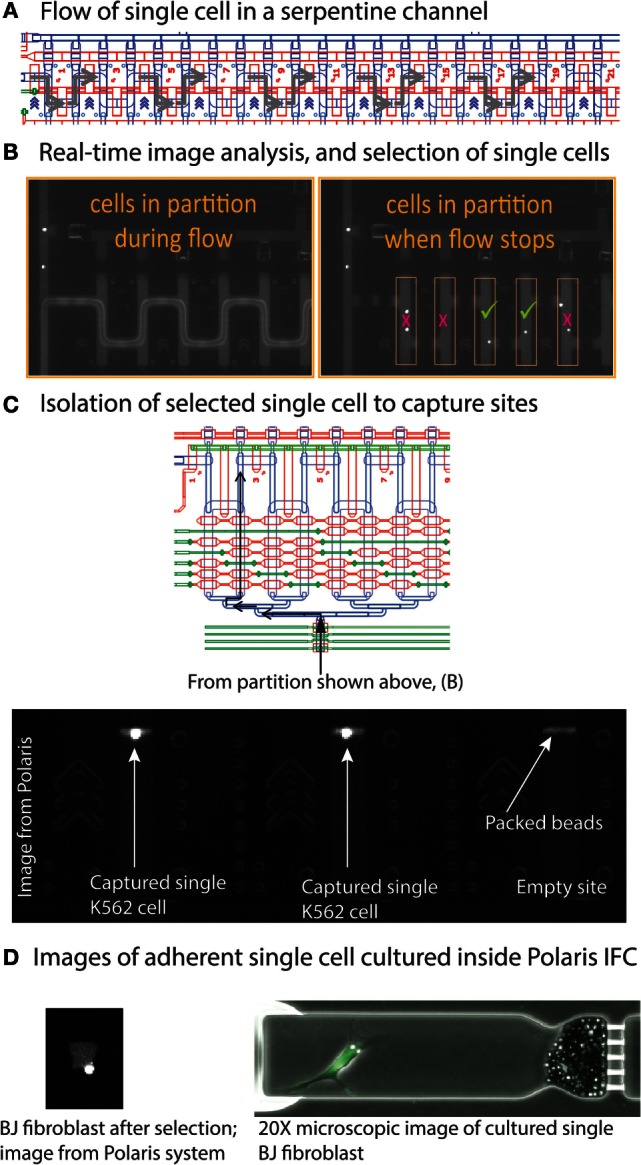
**(A)** Cells in suspension are loaded into a serpentine partition channel (CAD design image). **(B)** Image analysis shows movement of fluorescent cells inside the serpentine channel during reagent flow. Once the reagent flow is stopped, single cells are separated from each other. The system software identifies single cells based on desired fluorescent markers. **(C)** After identification, single cells are isolated by moving them to the capture sites through a fluidic multiplexer. **(D)** Image of cultured BJ fibroblast cells. The Polaris IFC can be imaged on a microscope to obtain high-resolution micrographs.

The Polaris IFC is first primed to fill the control lines on the fluidic circuit, load cell capture beads, and the inside of PDMS channels is blocked to prevent non-specific absorption/adsorption of proteins. In order to capture and maintain the single cells in the sites, the capture sites (48 sites) are preloaded with beads that are linked on-IFC to fabricate a tightly packed bead column during the IFC prime step. In the case of adherent cells, ECM is coated inside the cell capture chambers during prime step. After completion of the prime step, the cell mix (cells with suspension reagent) is loaded on the Polaris IFC and single CTO^+^/CD59^+^ cells are selected to capture sites. We extensively tested the performance of the Polaris IFC and system at three different cell purities (3, 10, and 50%). The cell purity is defined as the ratio of CTO^+^/CD59^+^ cells to CTO^+^/CD59^−^ cells. During the cell selection step, the suspended cells are loaded into the serpentine partition channel (Figure 3). Subsequently, the flow inside the partition channel is stopped (Figure 5A), and the cells are imaged in the partition channel for different fluorescent markers, as selected by the user. Based on automated image analyses by the system’s software, only single cells with the desired combination of fluorescent markers are selected and isolated to the cell capture site. Any doublets or single cells with undesired fluorescent combinations are not selected by the software for further experimentation (Figure 5B). The selected single cells are moved to capture sites through a multiplexer (Figure 5C). The system takes images of the capture sites to confirm the arrival of single cells from a particular position in the partition to a particular capture site number. Figure 5C shows a typical image from the Polaris system showing K562 single cells captured inside sites packed with a column of beads. The system will then select and isolate all available single cells from a partition fill as per desired fluorescent marker combination. Once it completes selection of candidate cells, the system refills the serpentine partition to look for more candidate single cells. The system repeats this process to select and isolate single cells until it fills all 48 capture sites.

If desired, the single cells can then be cultured in the capture sites. It is possible to culture either suspension (e.g., K562) or adherent (e.g., BJ fibroblast) cells. For adherent cells, extracellular matrix can be coated inside the capture site during the IFC priming step. Figure 5D shows a Polaris image of a cultured BJ fibroblast (adhered). Based on the experimental design, it is possible to dose these single cells and on-IFC-cultured single cells with drugs or other cell stimuli. Finally, the single cells are processed through template-switching mRNA-seq chemistry for full-length cDNA generation and preamplification on-IFC.

### Full-Length cDNA Generation

Preamplified full-length cDNA of selected single cells are generated on-IFC, and the amplicons are harvested through 48 different outlets. We used the SMARTer Ultra^®^ Low RNA Kit for Illumina Sequencing (Clontech^®^, 634936) to generate preamplified cDNA. The selected and sequestered single cells were lysed using Polaris cell lysis mixture. The 28-μL cell lysis mix consists of 8.0 μL of Polaris Lysis Reagent (Fluidigm, 101-1637), 9.6 μL of Polaris Lysis Plus Reagent (Fluidigm, 101-1635), 9.0 μL of 3′ SMART™ CDS Primer II A (12 μM, Clontech, 634936), and 1.4 μL of Loading Reagent (20X, Fluidigm, 101-1004). Synthetic RNA spikes can be optionally used with cell lysis mix. We typically use ArrayControl™ RNA spikes 1, 4, and 7 (Thermo Fisher Scientific, AM1780) to establish the functionality of RT and PCR on-IFC. We also use ERCC spikes at 1:50,000 dilution (final in lysis mix) for efficiency and quantification estimations. In order to implement synthetic RNA spikes, we thoroughly mix 96.5 μL of loading reagent with 2.5 μL of SMARTer Kit RNase Inhibitor (40 U/μL; Clontech, 634936) and subsequently add 1 μL of synthetic RNA spike to this spike mix. If RNA spike is used, then 1.4 μL of the loading reagent is replaced with the spike mix. The thermal profile for single-cell lysis is 37°C for 5 min, 72°C for 3 min, 25°C for 1 min, and hold at 4°C.

The 48-μL preparation volume for RT contains 1X SMARTer Kit 5X First-Strand Buffer (5X; Clontech, 634936), 2.5-mM SMARTer Kit Dithiothreitol (100 mM; Clontech, 634936), 1-mM SMARTer Kit dNTP Mix (10 mM each; Clontech, 634936), 1.2-μM SMARTer Kit SMARTer II A Oligonucleotide (12 μM; Clontech, 634936), 1-U/μL SMARTer Kit RNase Inhibitor (40 U/μL; Clontech, 634936), 10-U/μL SMARTScribe™ Reverse Transcriptase (100 U/μL; Clontech, 634936), and 3.2 μL of Polaris RT Plus Reagent (Fluidigm, 101-1366). All the concentrations correspond to those found in the RT chambers inside the Polaris IFC. The thermal protocol for RT is 42°C for 90 min (RT), 70°C for 10 min (enzyme inactivation), and a final hold at 4°C.

The 90-μL preparation volume for PCR contains 1X Advantage 2 PCR Buffer [not short amplicon (SA)] (10X, Clontech, 639206, Advantage^®^ 2 PCR Kit), 0.4-mM dNTP Mix (50X/10 mM, Clontech, 639206), 0.48-μM IS PCR Primer (12 μM, Clontech, 639206), 2X Advantage 2 Polymerase Mix (50X, Clontech, 639206), and 1X Loading Reagent (20X, Fluidigm, 101-1004). All the concentrations correspond to those found in the PCR chambers inside the Polaris IFC. The thermal protocol for preamplification consists of 95°C for 1 min (enzyme activation), five cycles (95°C for 20 s, 58°C for 4 min, and 68°C for 6 min), nine cycles (95°C for 20 s, 64°C for 30 s, and 68°C for 6 min), seven cycles (95°C for 30 s, 64°C for 30 s, and 68°C for 7 min), and final extension at 72°C for 10 min. The preamplified cDNAs are harvested into 48 separate outlets on the Polaris IFC carrier.

### qPCR Analysis on Biomark™

Harvested samples from Polaris IFCs were analyzed by qPCR using 96.96 Dynamic Array™ IFCs and the Biomark™ HD system from Fluidigm. Processing of the IFCs and operation of the instruments were performed according to the manufacturer’s procedures. For detection using the RNA expression and splice variant assays, a Master Mix was prepared consisting of 360-μL SsoFast™ EvaGreen^®^ Supermix with Low ROX (BioRad 172-5211) and 36-μL 20 × DNA Binding Dye Sample Loading Reagent (Fluidigm 100-5360), and 3.3 μL of this mix was dispensed to each well of a 96-well assay plate. Diluted harvest product (2.7 μL) was added to each well, and the plate was briefly vortexed and centrifuged. Following priming of the IFC in the IFC Controller HX, 5 μL of the sample + Master Mix were dispensed to each sample inlet of the 96.96 IFC. Five microliters of each 10 × assay [5 μM each primer, 1 × assay Loading Reagent (Fluidigm 100-5359)] were dispensed to each Detector Inlet of the 96.96 IFC. After loading the assays and samples into the IFC in the IFC Controller HX, the IFC was transferred to the Biomark HD, and PCR was performed using the thermal protocol GE Fast 96 × 96 PCR + Melt v2.pcl. This protocol consists of a thermal mix of 70°C, 40 min; 60°C, 30 s, hot start at 95°C, 1 min, PCR cycle of 30 cycles of 96°C, 5 s; 60°C, 20 s, and melting using a ramp from 60 to 95°C at 1°C/3 s. Data were analyzed using Fluidigm Real-Time PCR Analysis software using the Linear (Derivative) Baseline Correction Method and the Auto (Global) Ct Threshold Method. The data are exported as a.csv file into an Excel^®^ macro to compile and compare the data against in-house specifications.

## Results

### Performance Evaluation of Polaris IFC

In order to statistically evaluate the performance of the Polaris IFC, we designed and developed two performance tests: (1) total-RNA-based performance test (RNA PT) and (2) single-cell-based key performance test (KPT). Since single cells are heterogeneous, it would be difficult to evaluate the performance uniformity across 48 capture sites using a cell-based test method. Hence, we developed a 20-cell-equivalent total-RNA PT to evaluate and improve the performance of the Polaris IFC during the initial phase of the IFC development process.

#### Total RNA-Based Performance Test

**Figure 6:**
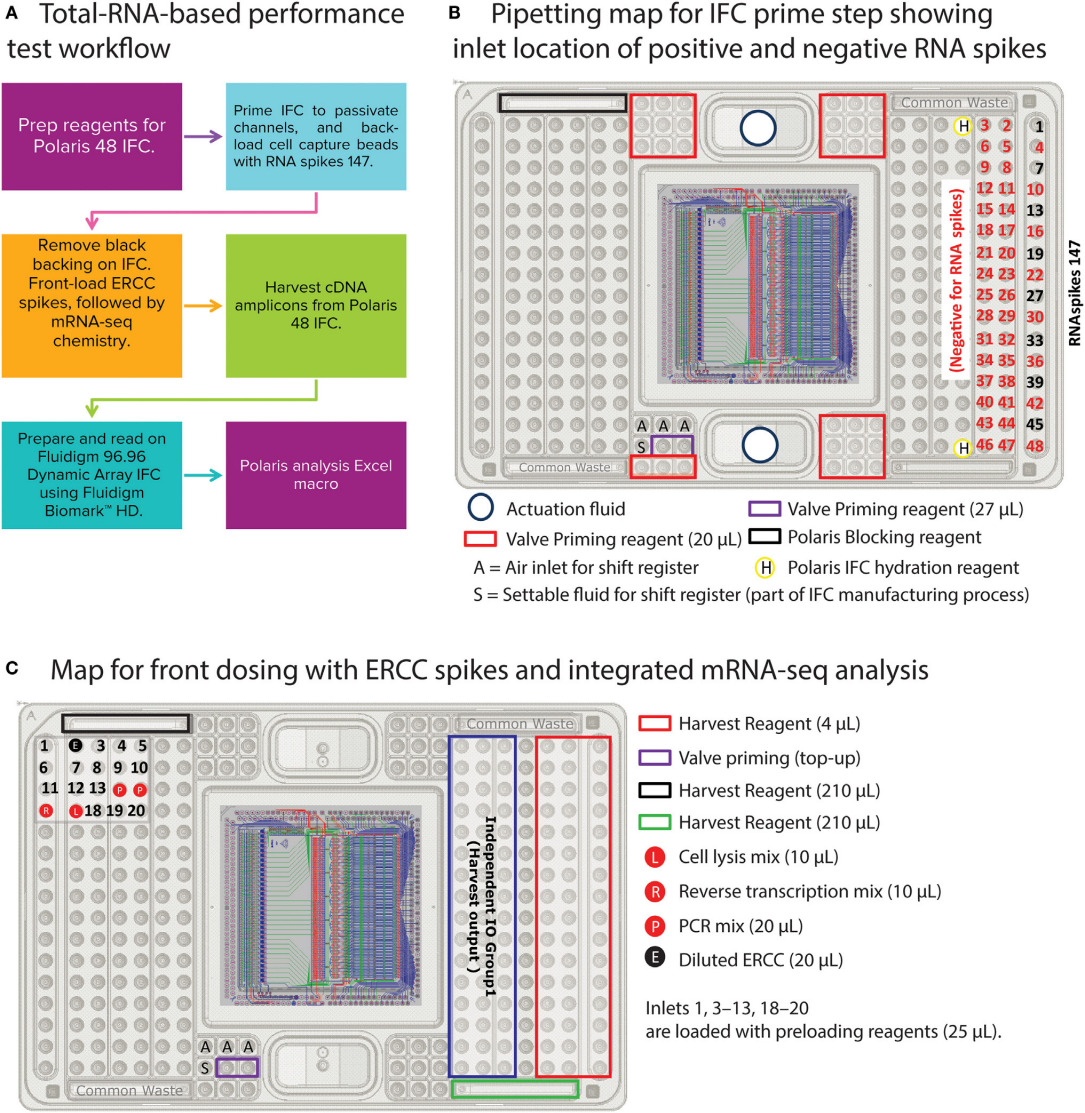
**Total-RNA-based performance test (two-step) workflow and pipetting map**. **(A)** Workflow to prime Polaris IFC with beads, back-load them with RNAspikes 147, simulate front dosing with diluted ERCC spikes, and generate cDNA using mRNA-seq chemistry. The cDNA amplicons from Polaris IFC were analyzed on the Fluidigm M96.96 IFC using 85 qPCR assays for specific genes, 8 assays for ERCC RNA spikes, and 3 assays for RNAspikes 147. **(B)** Pipetting map for IFC prime step. During prime step, RNAspikes 147 are back-loaded to 8 specific inlets with cell capture beads. **(C)** Pipetting map for front-dosing simulation with ERCC RNA spikes, followed by mRNA-seq chemistry.

The primary objective of this test is to statistically validate a workflow that is very close to the cell-based experiments on the Polaris system and yet collects critical information about uniformity of cDNA synthesis across IFC, reaction line cross-talk (on-IFC), and IFC-to-IFC correlation. To achieve this objective, we simulated steps such as loading of cell capture beads and the thermal step for cell lysis in the total-RNA PT. The workflow of the total-RNA PT is shown in Figure 6A. Briefly, the RNA-PT is a two-step procedure. In the first step, the control lines on the Polaris IFC are primed, channels are blocked, and cell capture beads are back-loaded with ArrayControl RNA SPIKES (1, 4, and 7 only, Thermo Fisher Scientific, AM1780; henceforth referred to as RNAspikes 147) in eight specific capture sites (Figure 6B).

**Figure 7:**
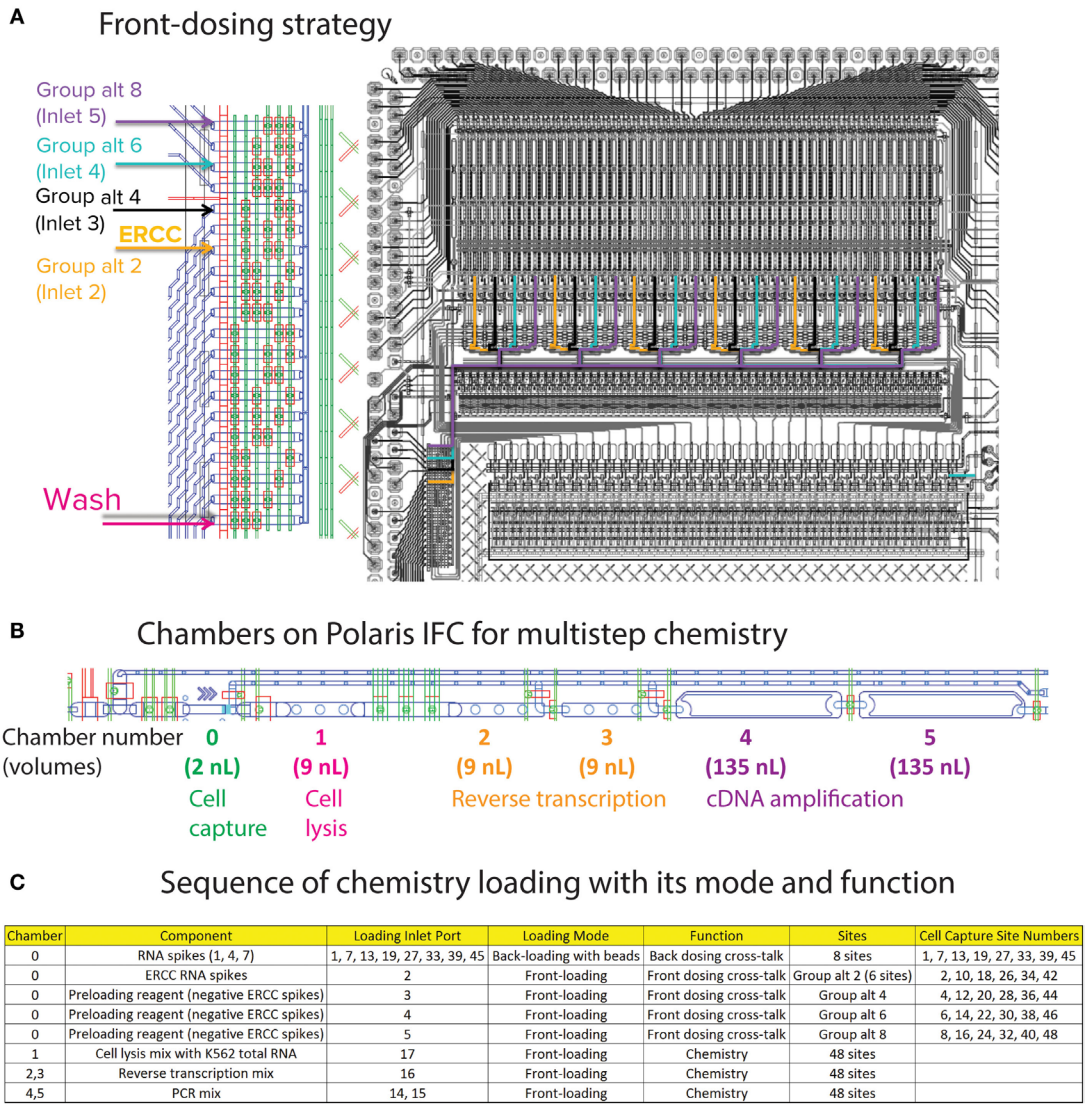
**(A)** Front-dosing strategy. The capture sites on Polaris IFC are segregated as six groups of eight sites. ERCC spike-in mix is loaded into the second capture site of every group (Group alt 2). For negative control, 1X Preloading Reagent was loaded into Group alt 4, 6, and 8. **(B)** Sequential chambers for multistep chemistry. The capture site with beads holds 2 nL of liquid. The lysis mix is loaded into chamber 1 (9 nL), then reverse transcription mix is loaded into chambers 2 and 3 (9 nL each), and PCR mix is loaded into chambers 4 and 5 (135 nL each). **(C)** Chemistry loading sequence and its function.

The ArrayControl RNA Spikes are used to evaluate the back-dosing cross-talk using highly sensitive qPCR assay designed to detect RNA spikes 1, 4, and 7 (three total ArrayControl RNA Spikes). After the priming step, six specific capture sites are loaded with ERCC RNA Spike-In Mix (Thermo Fisher Scientific, 4456740) to estimate the cross-talk for front-loaded reagents and dosing agents. Although the ERCC RNA mix contains 92 spike-ins, only 8 ERCC spike-ins were probed using qPCR assays. The front-dosing strategy is illustrated in Figure 7A and the pipetting map is shown in Figure 6C.

For negative control, 1X Preloading Reagent (Fluidigm, 100-9942) was loaded into specific inlets and capture sites (Figure 7C). After completion of front dosing, the mRNA-seq chemistry prep is integrated with the dosing step. The lysis mixture for the RNA-PT contains Leukemia (K562) Total RNA (Thermo Fisher Scientific, AM7832) at a concentration equivalent to 20 cells of total RNA in every cell capture site (48 sites). The cell capture site is serially connected to five chambers to enable multistep reaction chemistry (Figure 7B). Cell lysis mixture is loaded into the first 9-nL chamber. Then, RT mixture is loaded in 18-nL volume (two 9-nL chambers). Finally, PCR mixture for preamplification of full-length cDNA is loaded in 270-nL volume (two 135-nL chambers).

**Figure 8:**
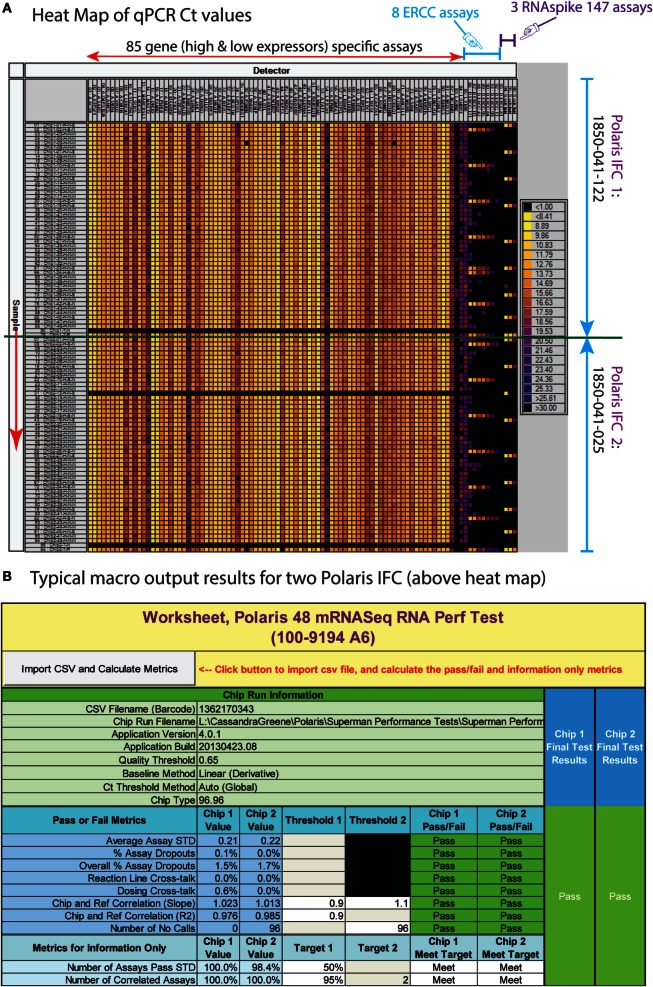
**(A)** Typical heat map of high-throughput qPCR assay for RNA-based performance test. The M96.96 IFC (96 samples) can accept amplicons from two Polaris IFCs (48 samples each). For every Polaris IFC, we replace two samples with positive and negative control samples. The columns are assays (85 high- and low-expressing assays; 8 ERCC spike assays; and 3 RNAspike 147 assays). The rows are diluted amplicons from Polaris IFCs. **(B)** Excel macro for the RNA-based performance test.

**Figure 9:**
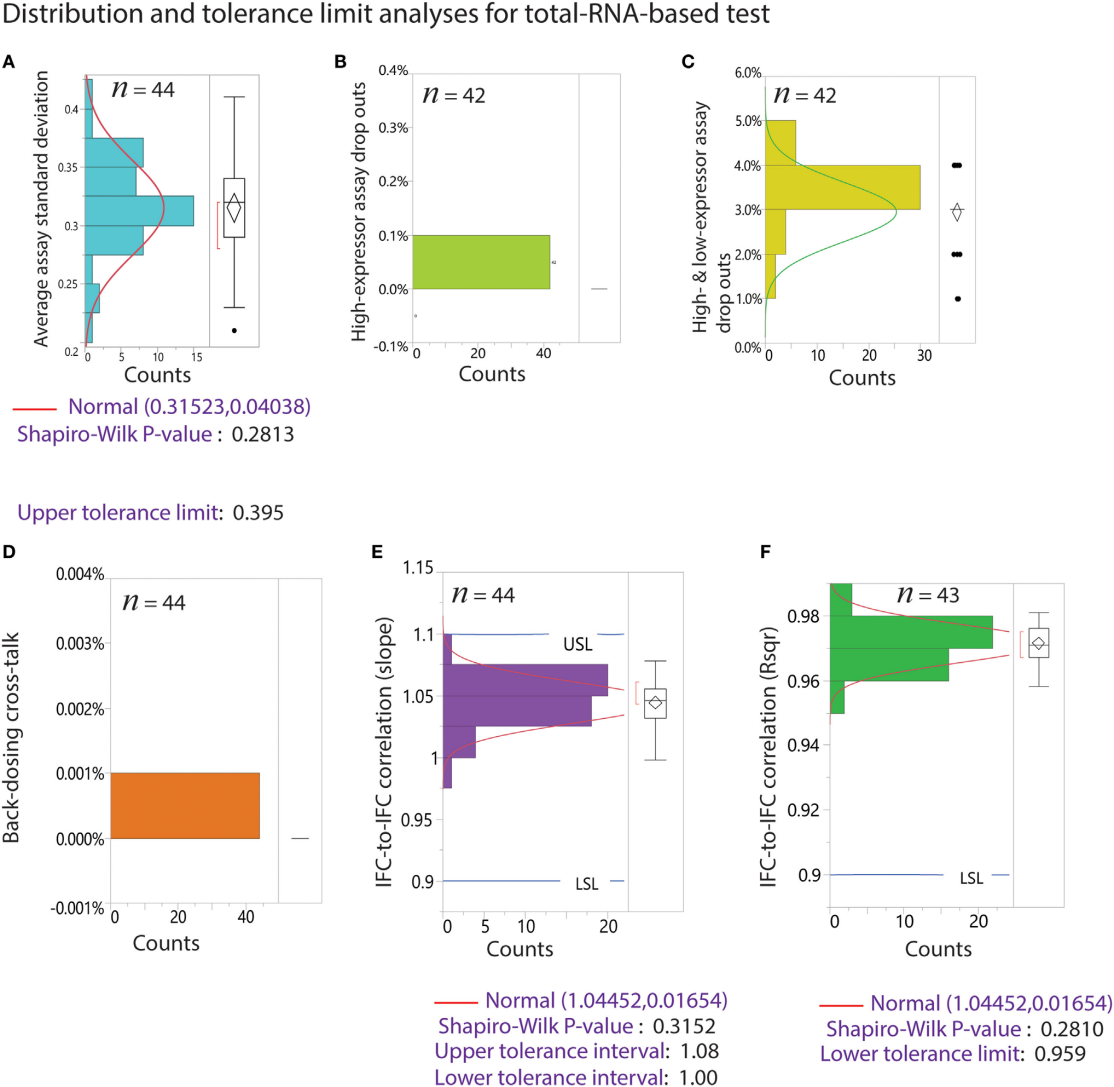
**Tolerance limit analyses from 44 Polaris IFC runs using RNA-based performance test**. *n* is the sample number after removal of outliers. Outliers are estimated by Tukey’s box plot. The percentage of outliers removed ranged from 0 to 5%. **(A)** Average assay SD. The data fit a normal distribution with goodness of fit *P*-value of 0.2813. The upper tolerance interval for this metric is 0.395. **(B)** Distribution of drop outs for high-expressing assays only. **(C)** Distribution of drop outs for both high and low expressors. **(D)** Back-loaded dosing cross-talk. **(E)** Distribution of slope for IFC-to-IFC correlation. The data fit a normal distribution with tolerance interval of 1–1.08. **(F)** Distribution of *R*^2^ for IFC-to-IFC correlation. The data fit a normal distribution with lower tolerance limit of 0.959.

The preamplified cDNA is harvested in ~7 μL volume. The harvest is further diluted by addition of 10 μL of DNA Dilution RGT (Fluidigm, 100-9167). In order to evaluate IFC uniformity and other performance metrics, we designed 88 Delta Gene™ assays (Fluidigm) for K562 (85 genes covering high and low expressors) and RNAspikes 147. In addition to these 88 assays, we used 8 ERCC qPCR assays from a published work (Devonshire et al., 2011). In total, we used 96 intercalating dye-based qPCR assays for read-out of RNA PT on an M96.96 Dynamic Array™ IFC (Fluidigm). We routinely test positive and negative tube controls for every chemistry preparation by qPCR assays on the M96.96. In order to do this, 2 out of the 48 samples from a Polaris IFC are replaced by positive and negative tube controls on M96.96. The positive tube control contains total RNA from K562, RNAspikes 147, and ERCC. The tube controls are used to validate the functionality of chemistry preparation on a particular day. Harvest products from two Polaris IFC are tested on a single M96.96 Dynamic Array IFC run. A typical qPCR Ct heat map and associated Excel macro for two Polaris RNA PTs are shown in Figures 8A,B. In order to statistically validate the performance, we tested 44 Polaris IFCs with RNA PTs. IFC and reagents from minimum of three manufacturing lots were used. Tolerance limit or interval analyses were performed on more than 40 Polaris IFC runs. The distribution of data and tolerance limit analyses for different metrics for the RNA PTs are shown in Figure 9.

#### Single-Cell-Based Key Performance Test

**Figure 10:**
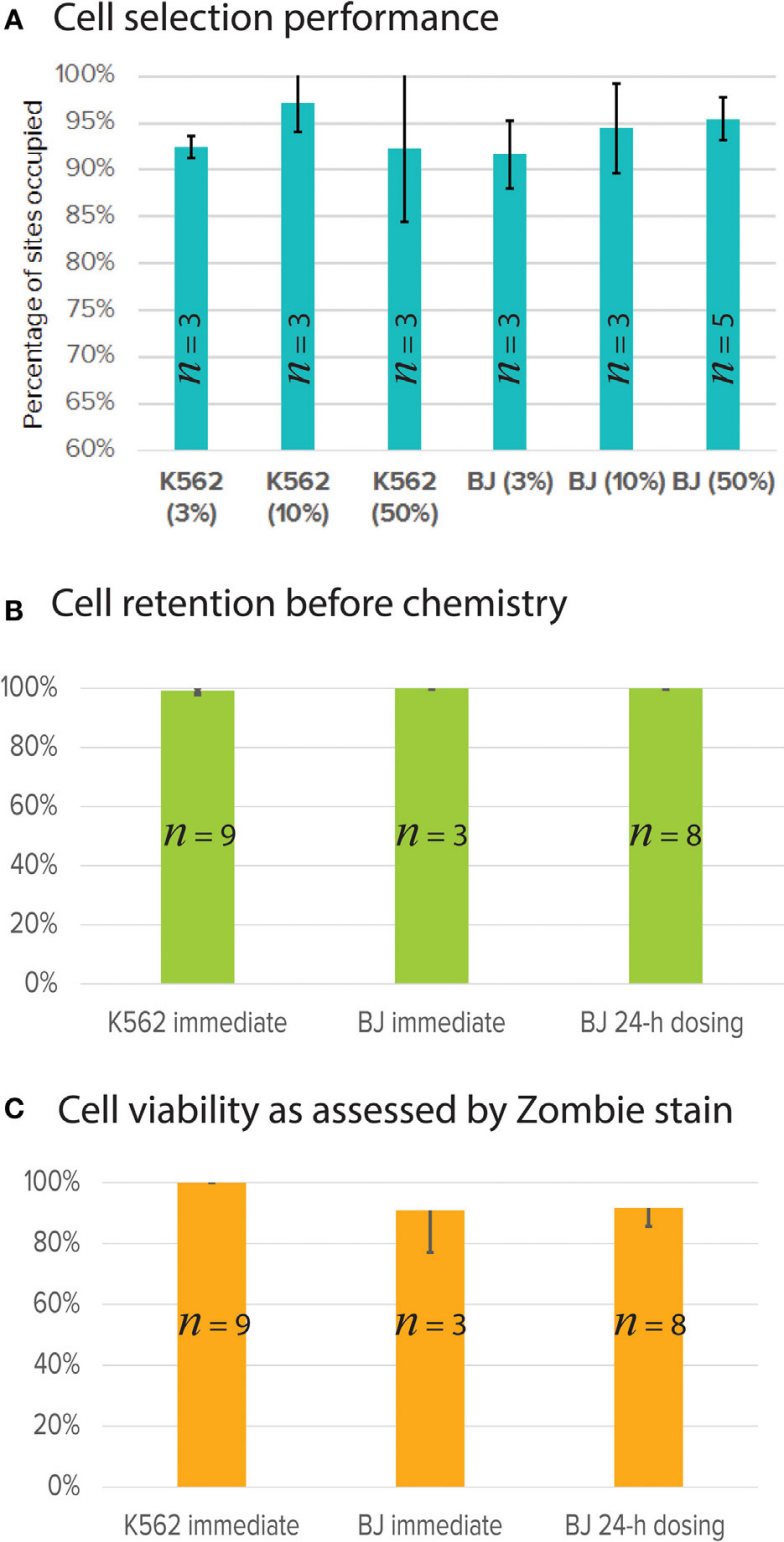
**Single-cell selection, retention, and viability performance using single-cell key performance test**. **(A)** Percentage of single cells selected (out of 48 total capture sites) for K562 (suspension) and BJ fibroblast (adherent) at different cell purity concentrations (3, 10, and 50%). *n* is number of Polaris IFCs tested. **(B)** Single cells retained after dosing and culture. Cells are imaged and counted before the cell lysis step. K562 and BJ fibroblasts were selected and immediately processed for mRNA-seq chemistry preparation. BJ fibroblasts were also selected, cultured, and dosed with cell culture medium for 24 h (BJ dosing). Data from three IFCs are presented here. **(C)** Single-cell viability as assessed by Zombie stain on-IFC.

Key performance test was developed and validated using one cell type each for suspension (K562) and adherent (BJ fibroblast) cells. As described in Section “Materials and Methods,” cells are stained with the universal fluorescent marker, CTO. A subset of these cells were stained for surface marker using antibody conjugated with Alexa 647. In the case of K562, we used Anti-Human CD59-Biotin (BD Biosciences, 555762) with Streptavidin Alexa 647, and for BJ fibroblast, we used Anti-Mouse/Human CD44-Alexa 647 (BioLegend 103018). The double-stained cells (universal CTO and surface marker Alexa 647) were mixed with cells stained with CTO only to achieve three different purity percentages (3, 10, and 50%). The cells were selected for universal CTO and surface marker. For BJ fibroblasts, we tested two different workflows, one with cell selection followed by chemistry (immediate) and another to dose the BJ fibroblasts with medium every 4 h for 24-h adherent culture, followed by chemistry (BJ dosing). In order to evaluate the cell viability prior to the cell lysis step, we used Zombie Yellow™ cell viability stain (BioLegend, 423103; λ_ex_ = 396 nm and λ_em_ = 572 nm), which stains dead cells. Performance metrics, such as number of sites occupied with single cells out of the total 48 sites (cell selection), number of cells retained after dosing and prior to cell lysis (cell retention), and number of viable cells prior to lysis, were evaluated. On average from 20 Polaris IFC runs, our cell selection was ~95% for K562 and BJ fibroblast with different purity percentages (Figure 10A). For cell retention, >47/48 sites showed presence of single cells as enumerated after the cell selection step and prior to cell lysis step (Figure 10B). The average cell viability was ~90% as estimated from 20 Polaris IFC runs (Figure 10C).

**Figure 11:**
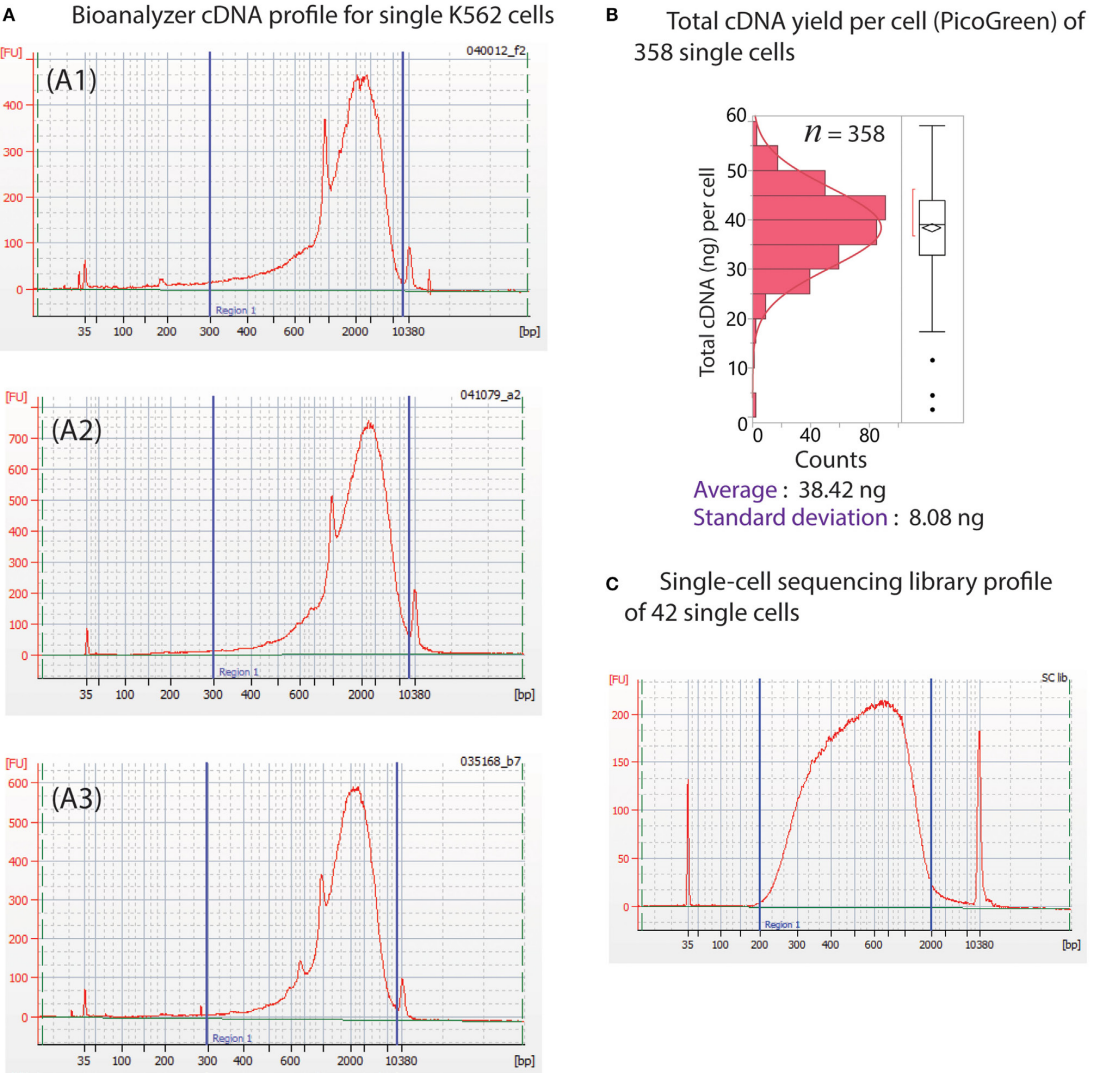
**(A)** Typical cDNA distribution from single cells from three different Polaris IFC runs (A1–A3). The peak for RNAspikes 147 is around 1,000 bp. **(B)** Distribution of cDNA yield from 358 single K562 cells from multiple Polaris IFC runs. The average cDNA yield per cell is 38.42 ng with a SD of 8.08 ng. **(C)** Library profile of 42 single cells.

**Figure 12:**
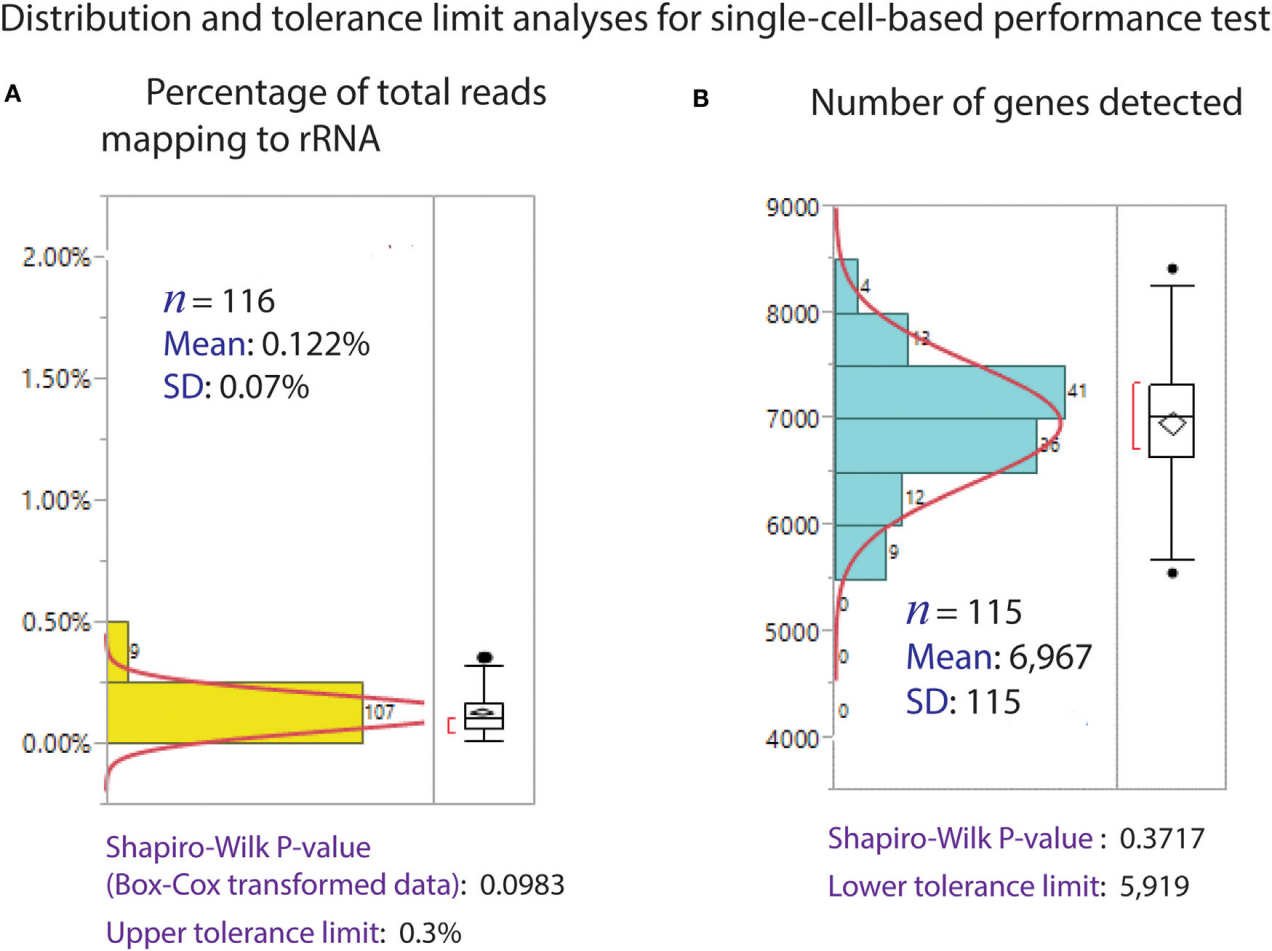
**Distribution of sequencing metrics for single cells**. **(A)** Percentage of reads mapped to rRNA/total reads. The average mapping to rRNA is 0.122% from 116 single-cell sequence datapoints. The upper tolerance limit for percentage rRNA mapping is 0.3%. **(B)** Number of genes detected (TPM > 1). Distribution of genes detected from 115 single-cell sequence data. The average genes detected is 6,967, with a SD of 115. The data fit a normal distribution, with a lower tolerance limit of 5,919.

The Polaris system generates very high quality (size distribution) and quantity (yield) of preamplified cDNA from single cells. The size distribution of preamplified cDNA from single cells, as evaluated using Bioanalyzer 2100 and the DNA high-sensitivity chip (Agilent), is typically in the size range of 0.3–7 kb (Figure 11A). For yield, preamplified cDNA from single cells was quantified using PicoGreen-based dsDNA quantification assay (Quant-iT™ PicoGreen^®^ dsDNA Assay Kit, Thermo Fisher Scientific, P7589). The average total cDNA yield per single K562 cell is 38.42 ± 8.08 ng (Figure 11B). We randomly selected ~14 single cells from three Polaris IFC runs, barcoded them using modified Nextera library prep, and pooled them to generate a single sequencing library. A representative library profile from 42 single cells is shown in Figure 11C. The majority of the single-cell library falls in the range of 200–2,000 bp. For three sequencing libraries from nine Polaris IFCs tested with K562 immediate chemistry, sequencing data from three MiSeq™ runs using v2 150 bp PE kit were compiled, and tolerance limits (90% confidence with 95% population coverage) were estimated for two key sequencing metrics (Figure 12). The average percentage of reads mapping to rRNA/total reads is 0.122%. The Box–Cox transformed data fit a normal distribution with a Shapiro–Wilk *P*-value of 0.0983. Based on the normal distribution, the upper tolerance limit for percentage of reads mapping to rRNA is 0.3% (Figure 12A). The mean number of genes detected is 6,967 ± 115. The data fit a normal distribution with a lower tolerance limit of 5,919 genes as estimated from 115 single-cell datapoints (Figure 12B).

**Figure 13:**
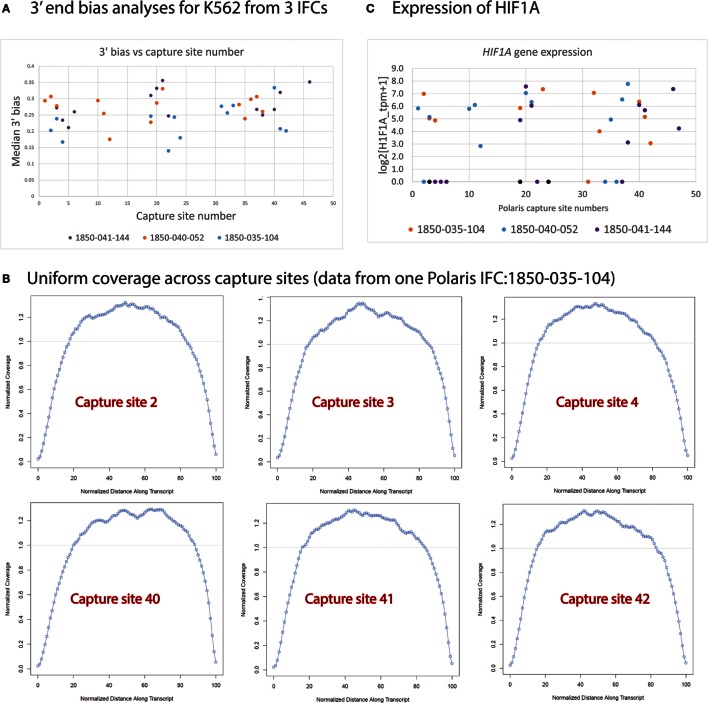
**Bias vs. capture site number**. **(A)** Median 3′ end bias was analyzed for random single cells picked for sequencing. No 3′ bias was noted for cells from three Polaris IFC runs **(B)** *HIF1A* gene expression of cells across different capture sites. **(C)** Sequencing coverage as estimated by Picard for single cells. Uniform coverage noted across different capture sites from a Polaris IFC.

We extensively analyzed our single-cell sequencing data for transcript coverage bias and possible positional bias of single cells selected across 48 capture sites on the Polaris IFC (Figure 13). We noted uniform coverage along the transcript length (Figure 13B) without any positional bias on the Polaris IFC. The plot of normalized coverage vs. normalized distance along the transcript with respect to capture sites (2, 3, 4 and 40, 41, 42) from a Polaris IFC is shown in Figure 13B. A plot of median 3′ end bias of transcript coverage with respect to capture site number indicates no positional bias across three Polaris IFC runs (Figure 13A). In order to understand if there is any possible effect of hypoxia on single cells due to spatial location of capture sites on the Polaris IFC, we analyzed the expression value of *HIF1A* gene across different capture sites. Up-regulation of hypoxia-induced factor 1 gene (*HIF1A*) is a known consequence of hypoxia (Choudhry and Mole, 2015). Expression analyses of *HIF1A* did not show any positional bias with respect to the capture sites (Figure 13C). It should be noted that we recommend strictly following the Polaris workflow as described in the Polaris protocol document (Fluidigm, 101-0082). Any deviation from the validated workflow might lead to introduction of possible bias at multiple levels.

### Sensitivity Studies Using ERCC Spike-Ins

**Figure 14:**
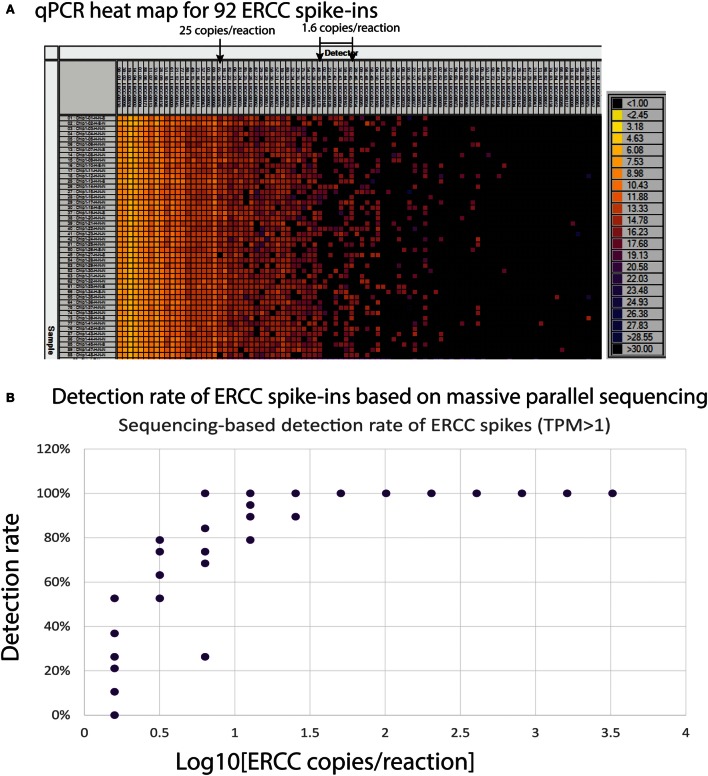
**(A)** Heat map (qPCR assay) for ERCC spike-in with single SKBR3 cells. The columns are 92 qPCR assays designed and developed for ERCC spike-in. The rows are amplicons from single SKBR3 cells with ERCC spike-in. The ERCC assays on the columns are sorted by concentrations. **(B)** Detection rate of ERCC spikes with >1.58 copies per Polaris IFC chambers.

An alternative way to evaluate performance of single-cell mRNA-seq on the Polaris system is to implement use of the ERCC RNA Spike-In Mix 1 in the lysis mix. The ERCC control mix consists of 92 polyadenylated transcripts with a size range of 273–2,022 bases and six orders of magnitude range in concentration. We tested both qPCR- and sequencing-based methods for detection of ERCC spikes. Ninety-two primer pairs were designed to target the corresponding transcripts for qPCR testing. qPCR was performed on a Fluidigm 96.96 Dynamic Array IFC. Stochastic distribution of transcripts was observed when the input concentration was 25 copies per reaction or less on the Polaris IFC followed by qPCR detection on the 96.96 Dynamic Array (Figure 14A). Single-copy RNA detection is demonstrated, although intermittently, likely due to sampling at the reaction site. Transcripts at 1.6 copies per reaction were intermittently detected by qPCR on the 96.96 Dynamic Array IFC. We also evaluated the detection rate of ERCC spikes (>1.6 copies/reaction) using an approach based on massive parallel sequencing. There were 7 ERCC spikes (ERCC-00170; ERCC-00148; ERCC-00126; ERCC-00099; ERCC-00054; ERCC-00163; ERCC-00059), which were at a concentration of 1.6 copies per Polaris reaction chamber. One of the 7 ERCC spikes (ERCC-00054) was not detected in any of the 19 single-cell samples. If we remove this datapoint as an outlier, the average detection rate of ~1.6 copies is 28%. Based on Poisson estimates, single-copy detection rate should be ~33% (67% should be a failure event). The single-copy detection rate (28%) from the Polaris system is very close to expected theoretical estimates based on Poisson statistics (Figure 14B).

### Single-Cell Transfection of nGFP mRNA and GFP Expression Analyses

**Figure 15:**
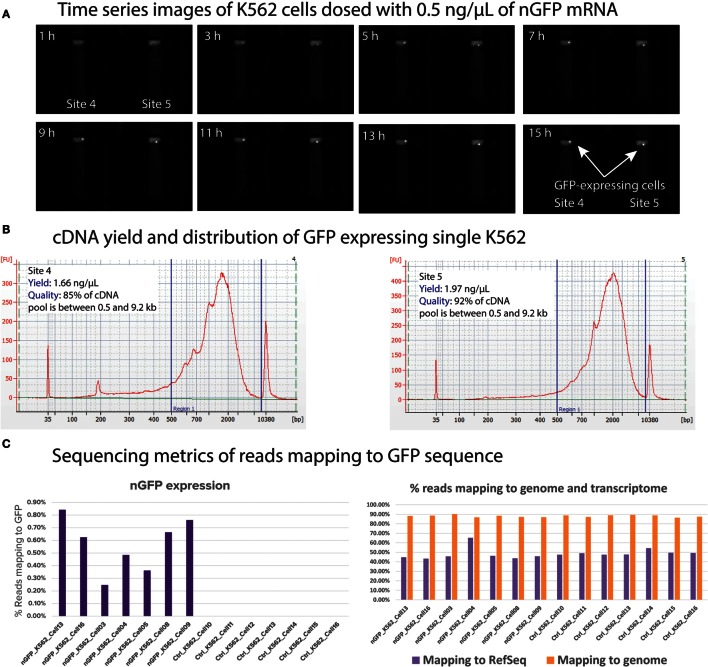
**(A)** Time-series images from the Polaris system taken every hour to monitor onset of GFP expression. For visual clarity, only two cell capture sites are shown. The onset GFP expression is noted around 3 h from the time of transfection. **(B)** cDNA yield and distribution from single K562 processed through mRNA-seq chemistry. **(C)** Mapping metrics of GFP-transfected single K562 cells to genome, transcriptome, and GFP sequence.

In order to demonstrate dosing and functional response analyses, we transfected single K562 cells with nuclear green fluorescent protein (nGFP) mRNA and cultured the transfected single K562 for 16 h. During this culture duration, the cells translated the nGFP mRNA and expressed the GFP inside the cell. The imaging capability of Polaris enables monitoring of GFP expression. Subsequently, the cells were processed for mRNA-seq chemistry on-IFC and sequenced on MiSeq to quantify the reads mapped to GFP. K562 cells stained with CTO were selected on Polaris IFC. To carry out the single-cell transfection, 10 μL of Stemfect RNA transfection reagent was mixed with 240-μL Stemfect transfection buffer (Stemgent^®^ Stemfect™ RNA Transfection Kit, 00-0069) (Mix A). The stock nGFP mRNA (Stemgent, 05-0019) at 100 ng/μL was diluted with Stemfect transfection buffer first and then further diluted with Mix A to make mRNA transfection complex. This complex was incubated at room temperature for 15 min and further diluted with K562 cell culture medium (refer to Section “K562 Cell Culture and CD59 Staining”) to achieve different final concentrations (0.5 and 1 ng/μL) of nGFP mRNA. Selected single K562 cells were cultured with the nGFP mRNA transfection complex with culture medium at 37°C with 5% CO_2_ on the Polaris IFC. During this cell culture incubation time, images were taken every hour to monitor the onset of GFP expression. Image analyses (Figure 15) showed that single cells picked up nGFP mRNA at 0.5 and 1 ng/μL concentrations and expressed the green fluorescent proteins, thereby reinforcing the fact that single cells on Polaris IFC are healthy and are able to uptake naked mRNA and translate it to protein capable of being transfected. Figure 15A shows typical time-series images, which can be obtained from the Polaris system. It should be noted that for this particular experiment, the imaging interval was set to 1 h, but the Polaris system is capable of taking successive images in a rapid mode. We noted onset of GFP gene expression around the 3-h time frame at single-cell resolution. The cDNA pool showed a length range from 0.3 to 9.2 kb, with an average length ~2 kb. It is also noted that >85% of the total cDNA pool lies between 0.5 and 9.2 kb (Figure 15B). Sequencing data show that the cells transfected with nGFP mRNA harbored the extracellular mRNA even after 16 h of culture. As expected, the control cells without nGFP transfection did not show any mapping to GFP sequence. The transfection of nGFP did not alter the mapping to genome and transcriptome when compared to the control cells (Figure 15C). The nGFP-transfected cells showed percentage average reads mapping of 0.57, 87.99, and 47.82% to GFP, genome, and transcriptome, respectively (*n* = 7), while the control K562 showed percentage average mapping of 0, 88.03, and 49.22 (*n* = 7).

## Discussion

In this work, we report design and development of an integrated system to perform functional studies on single cells. We developed a nanoscale IFC, which employs fluidic logic to actively select single cells, and a system capable of performing multiple functionalities. The performance of the developed IFC and system was extensively tested using RNA-based and single-cell-based performance tests. These tests were specifically designed to evaluate different functionalities of the IFC and system. The functional capability of the Polaris IFC and system has been successfully demonstrated using transfection of naked nGFP mRNA, followed by monitoring of nGFP expression and finally analysis of the whole set of mRNA transcripts by massive parallel sequencing. It is noted that it is not currently possible to perform studies reported in this work on any other single-cell platforms. The limitation of the current system includes limited number of cells for functional studies (up to 48 cells). However, the requirement on the number of cells depends on the biological question, and it is possible to expand the capability of the IFC consumable to process more cells in the future.

## Author Contributions

NR, BF, JS, and JAAW conceived and designed the RNA-based performance test; BF, LS, AAL, JS, and JAAW conceived and designed the single-cell-based performance test; NR, LS, AAL, JS, MLG, CDS, NDA, CG, CTL, IH, AO, CS, and JAAW performed experiments; BF, NSGKD, MZ, EO, and CP were involved in Polaris IFC development; KH, MTM, WY, MN, CC, ML, HC, ZH, LL, CC, and ZS were involved in Polaris system development; RY, WH, JA, and ZH were involved in Polaris software development; NR, LS, JS, CDS, XW, and JAAW analyzed the data; TS edited the manuscript; CL drafted the Polaris user guide; MU and JAAW supervised the project, helped with design and interpretation, and provided laboratory space and financial support; and NR, LS, KJL, and JAAW wrote the manuscript with input from all authors. All authors listed, have made substantial, direct and intellectual contribution to the work, and approved it for publication.

## Conflict of Interest Statement

All authors are employees of Fluidigm Corporation.

## References

[B1] Alix-Panabières C. Bartkowiak K. Pantel K. (2016). Functional studies on circulating and disseminated tumor cells in carcinoma patients. Mol. Oncol. 10, 443–449. 10.1016/j.molonc.2016.01.004 26847851 PMC5528980

[B2] Angermueller C. Clark S. J. Lee H. J. Macaulay I. C. Teng M. J. Hu T. X. (2016). Parallel single-cell sequencing links transcriptional and epigenetic heterogeneity. Nat. Methods 13, 229–232. 10.1038/nmeth.3728 26752769 PMC4770512

[B3] Avraham R. Haseley N. Brown D. Penaranda C. Jijon H. B. Trombetta J. J. (2015). Pathogen cell-to-cell variability drives heterogeneity in host immune responses. Cell 162, 1309–1321. 10.1016/j.cell.2015.08.027 26343579 PMC4578813

[B4] Bendall S. C. Simonds E. F. Qiu P. Ad A. Krutzik P. O. Finck R. (2011). Single-cell mass cytometry of differential immune and drug responses across a human hematopoietic continuum. Science 332, 687–696. 10.1126/science.1198704 21551058 PMC3273988

[B5] Bianconi E. Piovesan A. Facchin F. Beraudi A. Casadei R. Frabetti F. (2013). An estimation of the number of cells in the human body. Ann. Hum. Biol. 40, 463–471. 10.3109/03014460.2013.807878 23829164

[B6] Briggs S. F. Dominguez A. A. Chavez S. L. Reijo Pera R. A. (2015). Single-cell XIST expression in human preimplantation embryos and newly reprogrammed female induced pluripotent stem cells. Stem Cells 33, 1771–1781. 10.1002/stem.1992 25753947 PMC4441606

[B7] Cayrefourcq L. Mazard T. Joosse S. Solassol J. Ramos J. Assenat E. (2015). Establishment and characterization of a cell line from human circulating colon cancer cells. Cancer Res. 75, 892–901. 10.1158/0008-5472.CAN-14-2613 25592149

[B8] Choudhry H. Mole D. R. (2015). Hypoxic regulation of the noncoding genome and NEAT1. Brief. Funct. Genomics. 15, 174–185. 10.1093/bfgp/elv050 26590207 PMC4880005

[B9] Darmanis S. Gallant C. J. Marinescu V. D. Niklasson M. Segerman A. Flamourakis G. (2016). Simultaneous multiplexed measurement of RNA and proteins in single cells. Cell Rep. 14, 380–389. 10.1016/j.celrep.2015.12.021 26748716 PMC4713867

[B10] Devaraju N. S. G. K. Unger M. A. (2012). Pressure driven digital logic in PDMS based microfluidic devices fabricated by multilayer soft lithography. Lab. Chip 12, 4809–4815. 10.1039/c2lc21155f 23000861

[B11] Devonshire A. S. Elaswarapu R. Foy C. A. (2011). Applicability of RNA standards for evaluating RT-qPCR assays and platforms. BMC Genomics 12:118. 10.1186/1471-2164-12-118 21332979 PMC3052187

[B12] Dey S. S. Kester L. Spanjaard B. Bienko M. Oudenaarden A. (2015). Integrated genome and transcriptome sequencing of the same cell. Nat. Biotechnol. 33, 285–289. 10.1038/nbt.3129 25599178 PMC4374170

[B13] Ennen M. Keime C. Kobi D. Mengus G. Lipsker D. Thibault-Carpentier C. (2014). Single-cell gene expression signatures reveal melanoma cell heterogeneity. Oncogene 34, 3251–3263. 10.1038/onc.2014.262 25132268

[B14] Fan H. C. Fu G. K. Fodor S. P. A. (2015). Combinatorial labeling of single cells for gene expression cytometry. Science 347, 1258367 10.1126/science.1258367 25657253

[B15] Frei A. P. Bava F.-A. Zunder E. R. Hsieh E. W. Y. Chen S.-Y. Nolan G. P. (2016). Highly multiplexed simultaneous detection of RNAs and proteins in single cells. Nat. Methods 13, 269–275. 10.1038/nmeth.3742 26808670 PMC4767631

[B16] Gao D. Vela I. Sboner A. Iaquinta P. J. Karthaus W. R. Gopalan A. (2014). Organoid cultures derived from patients with advanced prostate cancer. Cell 159, 176–187. 10.1016/j.cell.2014.08.016 25201530 PMC4237931

[B17] Guo G. Pinello L. Han X. Lai S. Shen L. Lin T.-W. (2016). Serum-based culture conditions provoke gene expression variability in mouse embryonic stem cells as revealed by single-cell analysis. Cell Rep. 14, 956–965. 10.1016/j.celrep.2015.12.089 26804902 PMC4740311

[B18] Kim K.-T. Lee H. W. Lee H.-O. Kim S. C. Seo Y. J. Chung W. (2015). Single-cell mRNA sequencing identifies subclonal heterogeneity in anti-cancer drug responses of lung adenocarcinoma cells. Genome Biol. 16, 1–15. 10.1186/s13059-015-0692-3 26084335 PMC4506401

[B19] Klein A. M. Mazutis L. Akartuna I. Tallapragada N. Veres A. Li V. (2015). Droplet barcoding for single-cell transcriptomics applied to embryonic stem cells. Cell 161, 1187–1201. 10.1016/j.cell.2015.04.044 26000487 PMC4441768

[B20] Macaulay I. C. (2015). G&T-seq: parallel sequencing of single-cell genomes and transcriptomes. Nat. Methods 12, 519–522. 10.1038/nmeth.3370 25915121

[B21] Macosko E. Z. Basu A. Satija R. Nemesh J. Shekhar K. Goldman M. (2015). Highly parallel genome-wide expression profiling of individual cells using nanoliter droplets. Cell 161, 1202–1214. 10.1016/j.cell.2015.05.002 26000488 PMC4481139

[B22] Pollen A. A. Nowakowski T. J. Chen J. Retallack H. Sandoval-Espinosa C. Nicholas C. R. (2015). Molecular identity of human outer radial glia during cortical development. Cell 163, 55–67. 10.1016/j.cell.2015.09.004 26406371 PMC4583716

[B23] Saadatpour A. Guo G. Orkin S. H. Yuan G.-C. (2014). Characterizing heterogeneity in leukemic cells using single-cell gene expression analysis. Genome Biol. 15, 1–13. 10.1186/s13059-014-0525-9 25517911 PMC4262970

[B24] Shalek A. K. Satija R. Shuga J. Trombetta J. J. Gennert D. Lu D. (2014). Single-cell RNA-seq reveals dynamic paracrine control of cellular variation. Nature 510, 363–369. 10.1038/nature13437 24919153 PMC4193940

[B25] Unger M. A. Chou H.-P. Thorsen T. Scherer A. Quake S. R. (2000). Monolithic microfabricated valves and pumps by multilayer soft lithography. Science 288, 113–116. 10.1126/science.288.5463.113 10753110

[B26] Wilson N. K. Kent D. G. Buettner F. Shehata M. Macaulay I. C. Calero-Nieto F. J. (2015). Combined single-cell functional and gene expression analysis resolves heterogeneity within stem cell populations. Cell Stem Cell 16, 712–724. 10.1016/j.stem.2015.04.004 26004780 PMC4460190

[B27] Yu M. Bardia A. Aceto N. Bersani F. Madden M. W. Donaldson M. C. (2014). Ex vivo culture of circulating breast tumor cells for individualized testing of drug susceptibility. Science 345, 216–220. 10.1126/science.1253533 25013076 PMC4358808

