# Corrigendum: Fluidic Logic Used in a Systems Approach to Enable Integrated Single-Cell Functional Analysis

**DOI:** 10.3389/fbioe.2017.00011

**Published:** 2017-03-14

**Authors:** Naveen Ramalingam, Brian Fowler, Lukasz Szpankowski, Anne A. Leyrat, Kyle Hukari, Myo Thu Maung, Wiganda Yorza, Michael Norris, Chris Cesar, Joe Shuga, Michael L. Gonzales, Chad D. Sanada, Xiaohui Wang, Rudy Yeung, Win Hwang, Justin Axsom, Naga Sai Gopi Krishna Devaraju, Ninez Delos Angeles, Cassandra Greene, Ming-Fang Zhou, Eng-Seng Ong, Chang-Chee Poh, Marcos Lam, Henry Choi, Zaw Htoo, Leo Lee, Chee-Sing Chin, Zhong-Wei Shen, Chong T. Lu, Ilona Holcomb, Aik Ooi, Craig Stolarczyk, Tony Shuga, Kenneth J. Livak, Cate Larsen, Marc Unger, Jay A. A. West

**Affiliations:** ^1^New Technologies Research Department, Fluidigm Corporation, South San Francisco, CA, USA

**Keywords:** single-cell, mRNA-seq, functional studies, Fluidigm, Polaris

**Reason for Corrigendum:**

The first three authors Naveen Ramalingam, Brian Fowler, and Lukasz Szpankowski contributed equally to this work.

Cate Larsen (author) was missed in the original list.

**Corrected/new list**

Naveen Ramalingam^†^, Brian Fowler^†^, Lukasz Szpankowski^†^, Anne A. Leyrat, Kyle Hukari, Myo Thu Maung, Wiganda Yorza, Michael Norris, Chris Cesar, Joe Shuga, Michael L. Gonzales, Chad D. Sanada, Xiaohui Wang, Rudy Yeung, Win Hwang, Justin Axsom, Naga Sai Gopi Krishna Devaraju, Ninez Delos Angeles, Cassandra Greene, Ming-Fang Zhou, Eng-Seng Ong, Chang-Chee Poh, Marcos Lam, Henry Choi, Zaw Htoo, Leo Lee, Chee-Sing Chin, Zhong-Wei Shen, Chong T. Lu, Ilona Holcomb, Aik Ooi, Craig Stolarczyk, Tony Shuga, Kenneth J. Livak, Cate Larsen, Marc Unger and Jay A. A. West*

^†^Equal contribution.

The original article has been updated.

## Author Contributions

NR, BF, JS, and JAAW conceived and designed the RNA-based performance test; BF, LS, AAL, JS, and JAAW conceived and designed the single-cell-based performance test; NR, LS, AAL, JS, MLG, CDS, NDA, CG, CTL, IH, AO, CS, and JAAW performed experiments; BF, NSGKD, MZ, EO, and CP were involved in Polaris IFC development; KH, MTM, WY, MN, CC, ML, HC, ZH, LL, CC, and ZS were involved in Polaris system development; RY, WH, JA, and ZH were involved in Polaris software development; NR, LS, JS, CDS, XW, and JAAW analyzed the data; TS edited the manuscript; CL drafted the Polaris user guide; MU and JAAW supervised the project, helped with design and interpretation, and provided laboratory space and financial support; and NR, LS, KJL, and JAAW wrote the manuscript with input from all authors. All authors listed, have made substantial, direct and intellectual contribution to the work, and approved it for publication.

## Conflict of Interest Statement

All authors are employees of Fluidigm Corporation.

